# Geographic diversity of the *Streptococcus equi* subsp. *equi* accessory genome: implications for vaccines and global surveillance

**DOI:** 10.3389/fvets.2025.1721958

**Published:** 2025-11-28

**Authors:** Lingyu He, Nwai Oo Khine, Jeongmin Song, Celine Loubière, Patrick Butaye

**Affiliations:** 1 Jockey Club College of Veterinary Medicine and Life Sciences, City University of Hong Kong, Kowloon, Hong Kong SAR, China; 2 Department of Microbiology and Immunology, Cornell University College of Veterinary Medicine, Cornell University, Ithaca, NY, United States; 3 Faculty of Veterinary Medicine, Department of Pathobiology, Pharmacology and Zoological Medicine, Ghent University, Merelbeke, Belgium

**Keywords:** horse, strangles, virulence-associated genes, antibiotic resistance genes, mobile genetic elements

## Abstract

Strangles, caused by the host-adapted *Streptococcus equi* subsp. *equi* (*S*. *equi*), imposes significant welfare and economic losses on the equine industry worldwide. Understanding its genomic features, virulence-associated genes (VAGs), antimicrobial resistance (AMR) and mobile genetic elements (MGEs) is essential for disease control and vaccine development. This study aimed to characterize the accessory genome composition, geographic distribution of VAGs and MGEs, and AMR profiles of *S*. *equi* by a large-scale genomic analysis of global publicly available *S*. *equi* sequences. All publicly available *S*. *equi* sequences in the Sequence Read Archive (SRA) database were retrieved and assembled. A total of 552 high-quality assemblies were obtained for further analysis. The strains originated from five continents (North/South America, Europe, Asia and Oceania). The geographical distribution of VAGs (analyzed using an in-house *Streptococcus equi* virulence factor database), antibiotic resistance gene (ARG) profiles, and the contribution of MGEs to *S*. *equi* VAGs were analyzed in this study. The results revealed that *S*. *equi* exhibited a closed pangenome with 1,661 core and 982 accessory genes. Among 71 identified VAGs, 40 were core VAGs, while accessory VAGs showed significant geographic variations, especially in nutritional/metabolic factor genes and exotoxin genes. No acquired ARGs were detected except a single *qacG* gene encoding resistance to quaternary ammonium compounds. This study revealed a functional specialization of MGEs, where prophages carry superantigen genes (*speH*, *speI*) and the hyaluronidase gene *hylP*; genomic islands (GIs) harbor iron acquisition genes (*eqb* cluster) and the *virD4* gene encoding the T4SS coupling protein; and integrative conjugative elements (ICEs) carry the heme metabolism cluster (*htsA*, *shp*) and streptolysin S-associated genes (*sagA*, *sagD*). The geographic variation of VAGs suggests regional adaptive pressures and supports genome streamlining in *S*. *equi*. In conclusion, *S*. *equi* exhibits a closed and streamlined genome, characteristic of host-adapted bacteria. There is a minimal acquisition of ARGs while key VAGs are retained. Prophages, GIs, and ICEs play specialized roles in VAG distribution. These findings provide insights into prioritizing VAGs for strangles vaccine development and surveillance of antigenic variation to mitigate vaccine escape.

## 1 Introduction

Strangles, caused by *Streptococcus equi* subspecies *equi* (*S*. *equi*), is a widespread infectious disease of horses that poses significant welfare and economic costs worldwide (1). The disease is characterized by pyrexia, acute swelling and subsequent abscess formation of the submandibular and retropharyngeal lymph nodes (2). Approximately 10% of recovered horses become persistent carriers harboring *S*. *equi* in the guttural pouches without clinical signs of disease. These are so-called “silent carriers” (3). *S*. *equi* is a host-adapted *β*-hemolytic bacterium of the Lancefield group C streptococcal that evolved from the broader-host-range subspecies *Streptococcus equi* subspecies *zooepidemicus* (*S*. *zooepidemicus*) (4). *Streptococcus equi* subspecies share >80% DNA sequence homology with Lancefield group A *Streptococcus pyogenes* (*S*. *pyogenes*) (5), a major human pathogen. Genomic studies have revealed that *S*. *equi* has undergone significant genome specialization and decay, driven by its persistent infection in horses (6). This evolution toward host restriction was characterized by the loss and acquisition of various genetic elements, especially virulence-associated genes (VAGs) and mobile genetic elements (MGEs) (6).

The multicomponent subunit vaccine against strangles, Strangvac, is composed of eight antigens (7). These components are CNE, a collagen-binding protein that mediates adhesion to the host extracellular matrix (8); EAG, which binds host proteins like IgG and albumin for immune evasion (9); three collagen-like proteins (SclC, SclF, SclI) that are immunogenic during infection (10); two surface proteins, SEQ_0402 and SEQ_0256, with SEQ_0402 (Eq8) being implicated in virulence (11); and IdeE, a secreted IgG-degrading enzyme that disrupts antibody-mediated immunity (12). In addition to these, other VAGs are critical to *S*. *equi* pathogenicity. One of the key virulence factors of *S*. *equi* is the M-like protein (SeM) encoded by *fbp*, a primary cell wall-associated protein and a protective antigen (13). The *fbp* gene has also been used as a target for the molecular detection and differentiation of *S*. *equi* from its closely related subspecies *S*. *zooepidemicus* (14). Furthermore, other VAGs in the accessory genome of *S*. *equi*, such as the superantigen-encoding genes *speH* (*seeH*), *speI* (*seeI*), *speK* (*seeL*), and *speL* (*seeM*), are important to the ability of this host-restricted pathogen to cause lymph node abscess and strangles (5, 15). A deeper understanding of the distribution and function of these diverse virulence factors is therefore essential for advancing vaccine development and disease surveillance.

Although previous studies have characterized the core genome of *S*. *equi* strains from 18 countries (16), there is no complete database of all VAGs of *S*. *equi*, leaving critical gaps in the geographic variations of its accessory genome. In addition, the evolutionary pressure causing accessory genome adaptation and the geographical variations have not been systematically investigated. Therefore, a comprehensive analysis of 552 high-quality *S*. *equi* genomes spanning five continents (1965–2023) was conducted. Our study reveals the accessory genome structure of *S*. *equi* and provides valuable information for vaccine design strategies and acquired antimicrobial resistance (AMR) surveillance. The analysis of the accessory genome of *S*. *equi* provides additional insights into the genomic decay and specialization model in this host-adapted pathogen (6).

## 2 Materials and methods

### 2.1 Strain collection, genome assembly and pan-genome analysis

Publicly available *S*. *equi* whole genome sequencing raw read samples (n = 985) were downloaded from the Sequence Read Archive (SRA) database of National Center for Biotechnology Information (NCBI) in July 2024 with keywords “*Streptococcus equi* subsp. *equi*.” The complete genome of *Se*4047, isolated from a case of strangles in the UK in 1990, was used as the reference (5). *De novo* assembly was conducted using the normal mode of Unicycler (v0.5.0) with default parameters (17), quality was assessed with QUAST (v5.2.0) (18). Genome assembly metrics derived from QUAST were obtained for all strains to validate genome quality. To ensure high-quality assemblies for downstream analysis, stringent quality control criteria were applied. Assemblies were excluded if they met any of the following criteria: (i) N50 < 30,000 bp, (ii) NG50 < 30,000 bp, (iii) number of contigs > 200, (iv) total assembly length < 2,000,000 bp, (v) longest contig length < 80,000 bp, or (vi) genome fraction < 90% (19). These thresholds were established to remove assemblies with poor contiguity, excessive fragmentation, or incomplete genome coverage. The genome sequences were annotated using Prokka (v1.14.6) (20). The analysis was run specifying the genus as *Streptococcus*, enabling the use of genus-specific database, and providing the custom in-house *Streptococcus*_VFDB as an external protein source. The “metagenome” mode was also used to account for potential gene fragments at contig ends. The resulting GFF files were used for pan-genome analysis with Roary (v3.12.0) to determine the core and accessory genome components (21). Roary was executed with MAFFT for core gene alignment, disabling paralog splitting, and using a default BLASTp identity threshold. Core genes were defined as genes present in ≥99% of genomes, soft core genes present in 95–99% of genomes, shell genes present in 15–95% of genomes, and cloud genes present in <15% of genomes. Pan-genome accumulation curves for 552 *S*. *equi* genomes were generated from 100 random permutations. To quantitatively assess its nature, the mean pan-genome curve was fitted to a power-law function (P(n) = c * n^*γ*, P(n) is the pan-genome size for n genomes.) using the non-linear least squares (nls) function in R. The pan-genome was considered closed if the growth exponent γ was close to zero.

### 2.2 *Streptococcus_VFDB database* construction and VAGs detection

Putative VAGs in *Streptococcus equi* subspecies were systematically identified through a dual approach. First, a comprehensive literature review was conducted to curate a list of potential VAGs reported in *S*. *equi* and its closely related pathogen *S*. *zooepidemicus*. The literature search was performed using PubMed and Google Scholar databases up to November 2024. Search term was “*Streptococcus equi*.” All original research articles, reviews, and relevant genomic studies reporting genes implicated in pathogenicity were considered. Second, the Virulence Factor Database entries (VFDB)^1^ for the genus *Streptococcus* were included. The combined list was formatted into a custom BLAST database (hereafter referred to as the in-house *Streptococcus_*VFDB) as a search-ready BLAST database for ABRicate (v1.0.1; see Supplementary Table S1 for the complete list of curated VAGs in the in-house *Streptococcus*_VFDB database).^2^ VAG presence was assessed using ABRicate with default alignment criteria (with ≥80% identity and ≥80% coverage).

### 2.3 Identification of ARGs

Acquired ARGs and chromosomal mutations known to confer resistance were identified using ABRicate (v1.0.1) with the ResFinder database (March 2025) with default parameters and the Comprehensive Antibiotic Resistance Database—Resistance Gene Identifier (CARD-RGI) (v6.0.3) tool (minimum identity ≥95%) (22).

### 2.4 Identification of MGEs

Prophage regions were detected using PHASTEST (v3.0) (23). Integrative conjugative elements (ICEs) and integrative mobilizable elements (IMEs) were identified using ICEscreen (v1.3.2) and ICEfinder2 (v2.0) (24, 25). BLAST comparisons were performed against all known ICE sequences from the ICEberg database (v2.0) to identify and classify the detected ICE elements (26). Genomic islands (GIs) were identified using IslandViewer4 (27), which incorporates IslandPath-DIMOB (28), IslandPick (27) and SIGI-HMM (29). Regions identified by at least two algorithms were recognized as a GI. All the bioinformatics tools were used with default parameters.

### 2.5 Prophage sequence similarity analysis

To refine the characterization of prophages detected in the assembled *S*. *equi* draft genomes, the nucleotide sequences identified by PHASTEST (v3.0) were aligned against reference sequences of known prophages ΦSeq1, ΦSeq2, ΦSeq3, and ΦSeq4 from the *S*. *equi* Se4047 genome (NCBI Reference Sequence: NC_012471.1). Pairwise global alignments were performed using the Needleman-Wunsch algorithm as implemented in the EMBOSS needle tool (v6.6.0.0) with default parameters (30). Nucleotide identity percentages were calculated to assess sequence similarity and aid in classifying prophages within the *S*. *equi* collection in the present study.

### 2.6 Identification of MGE-associated ARGs and VAGs

The sequences corresponding to identified prophages and ICEs were extracted from the assembled genomes using custom Python scripts. Comparative analysis to identify MGEs carrying ARGs and VAGs was performed by BLAST searching MGE draft sequences against the CARD database in CARD-RGI tool (v6.0.3; ≥ 80% identity) and in-house *Streptococcus*_VFDB databases [BLASTn (dc-megablast), ≥ 80% identity and ≥ 80% coverage], respectively.

### 2.7 Statistical analysis

Statistical analysis and visualization were performed using R (version 4.4.2) with the FSA (v0.9.6), car (v3.1.3), dunn.test (v1.3.6), multcompView packages (v0.1.10), ggplot2 (v3.5.1) and ComplexHeatmap (v2.22.0). The heat maps were drawn using ChiPlot.^3^ For comparisons among multiple groups, data were first assessed for normality using the Shapiro-Wilkinson test and for homogeneity of variances using Levene’s test. The Kruskal-Wallis rank sum test was used to evaluate differences across groups that had a non-normal distribution. When the Kruskal-Wallis test indicated statistically significant differences (*p* < 0.05), *post-hoc* pairwise comparisons were conducted using Dunn’s test with Bonferroni correction to adjust for multiple testing. Adjusted *p*-values were used to determine statistical significance, with thresholds defined as follows: *p* < 0.05 (*), *p <* 0.01 (**), and *p <* 0.001 (***). Bar charts display means with error bars representing 95% confidence intervals (CIs).

## 3 Results

### 3.1 Sequences included and geographic distribution of *S. equi*

**Figure 1:**
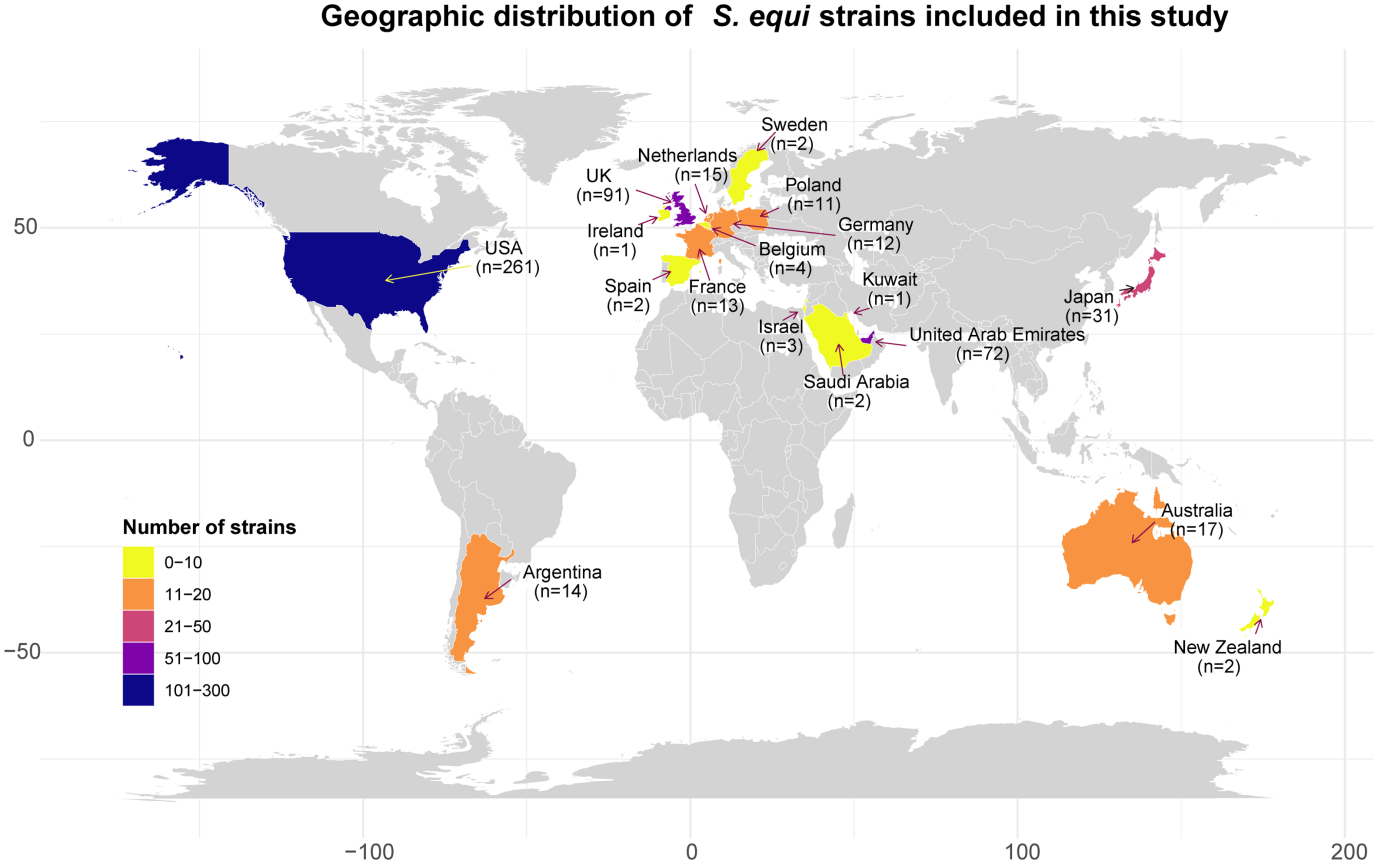
The geographical distribution of 552 *S*. *equi* strains examined in this study. Countries are colored according to the number of strains. The x-axis and y-axis represent longitude and latitude, respectively.

From the 985 *S*. *equi* genome sequences in the SRA database of NCBI (collected from 1965 to 2023), 552 high-quality *S*. *equi* assemblies could finally be included in the study. Detailed metadata for these 552 strains are present in Supplementary Table S2. The strains originated from 18 countries across five continents: North America (*n* = 261), Europe (*n* = 149), Asia (*n* = 109), Oceania (*n* = 19), and South America (*n* = 14; Figure 1).

### 3.2 Genomic features of *S. equi* and pangenome analysis

**Figure 2:**
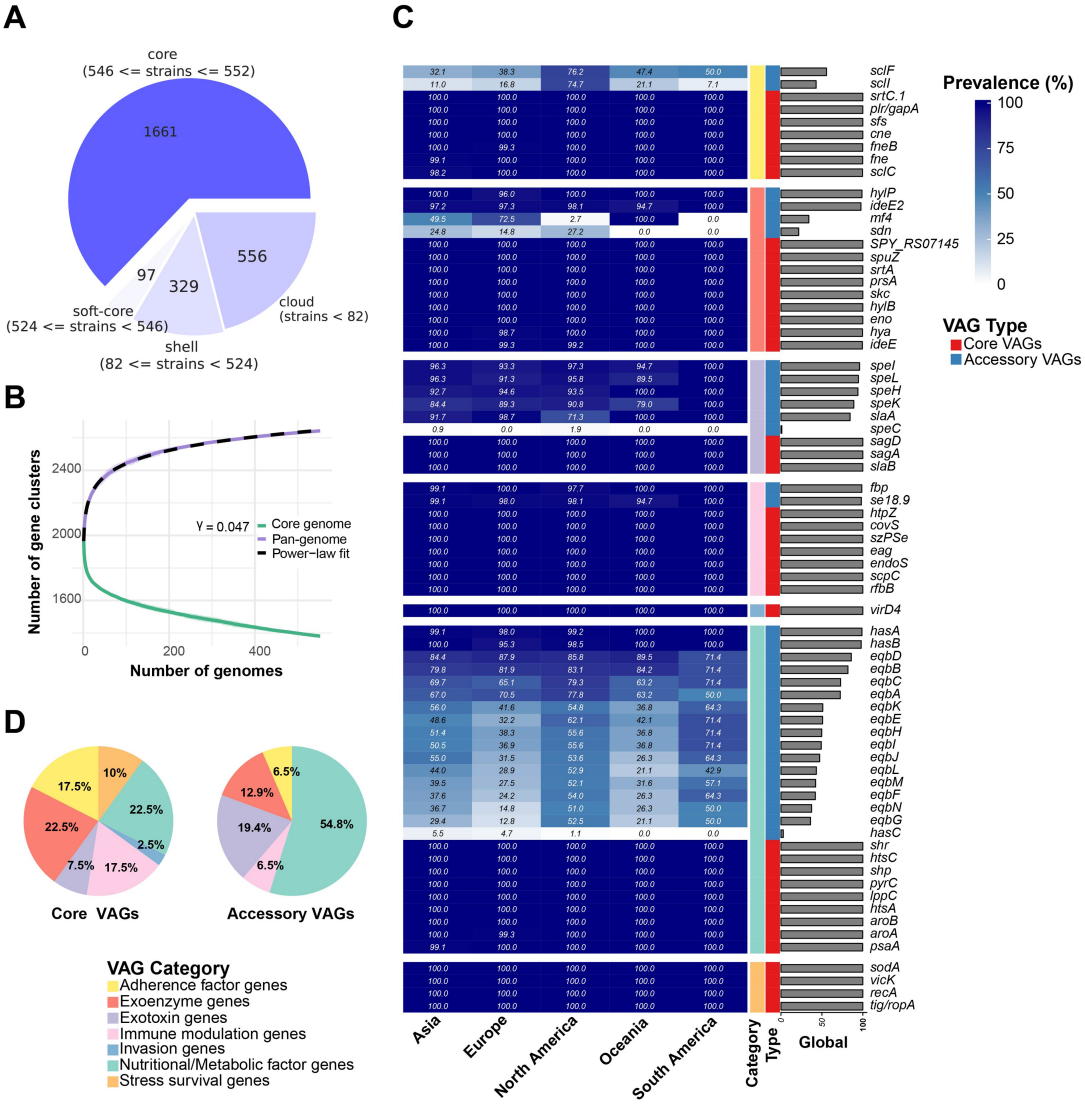
Pangenome analysis and VAG distribution pattern overview. **(A)** Pangenome analysis of 552 *S*. *equi* strains. The pie chart shows the number of protein-coding genes in the core, soft-core, shell, and cloud of the pangenome of 552 *S*. *equi* strains. The core genome is defined as genes present in 99–100% of 552 *S*. *equi* strains. Soft core, shell and cloud genomes are defined as those present in 95–99%, 15–95% and 0–15% of the strains, respectively. **(B)** Pan- and core-genome accumulation curves. The core-genome curve (green) and pan-genome curve (purple) show the number of gene clusters as genomes are sequentially added. The pan-genome curve was fitted to a power-law model (dashed line), yielding a *γ* of 0.047. Shaded areas indicate the interquartile range from 100 permutations. **(C)** Accessory VAGs prevalence in each continent and at the global level. **(D)** Different categories of core and accessory VAG proportion.

The average size and GC content were 2,088,082 bp and 41.3%, respectively (Supplementary Table S3). Pan-genome analysis revealed 1,661 core genes and 982 accessory genes (Figure 2A). The pan-genome accumulation curve rapidly reached a plateau, and yielded a growth exponent (*γ*) of 0.047 (Figure 2B), indicating *S*. *equi* possesses a closed pan-genome.

### 3.3 Accessory VAGs displayed geographical variation

A total of 71 VAGs in the 552 *S*. *equi* genomes were found (Figure 2C; Supplementary Table S4). Functional annotation and clustering of these 71 VAGs classified them into seven distinct functional groups: nutritional/metabolic factor genes, exoenzyme genes, immune modulation genes, exotoxin genes, adherence factor genes, stress survival genes, and invasion factor genes. Among these, 40 VAGs were core VAGs, being present in more than 99% of strains across all functional groups. All invasion genes (*n* = 1) and stress survival genes (*n* = 4) were core VAGs. The hyaluronate lyase encoding gene *hylP*, the IgG endopeptidase encoding gene *ideE2*, the superantigen encoding genes *speI*, *speL*, *speK*, and *speH*, the H factor binding protein encoding gene *se18*.*9*, the M-like protein SeM encoding gene *fbp*, and the hyaluronic acid capsule biosynthesis components encoding *has* operon genes *hasA* and *hasB* were largely conserved (Figure 2C). A total of 31 VAGs were less present and were part of only five functional groups, most of them were nutrition/metabolic factor genes (54.8%, 17/31; Figure 2D).

**Figure 3:**
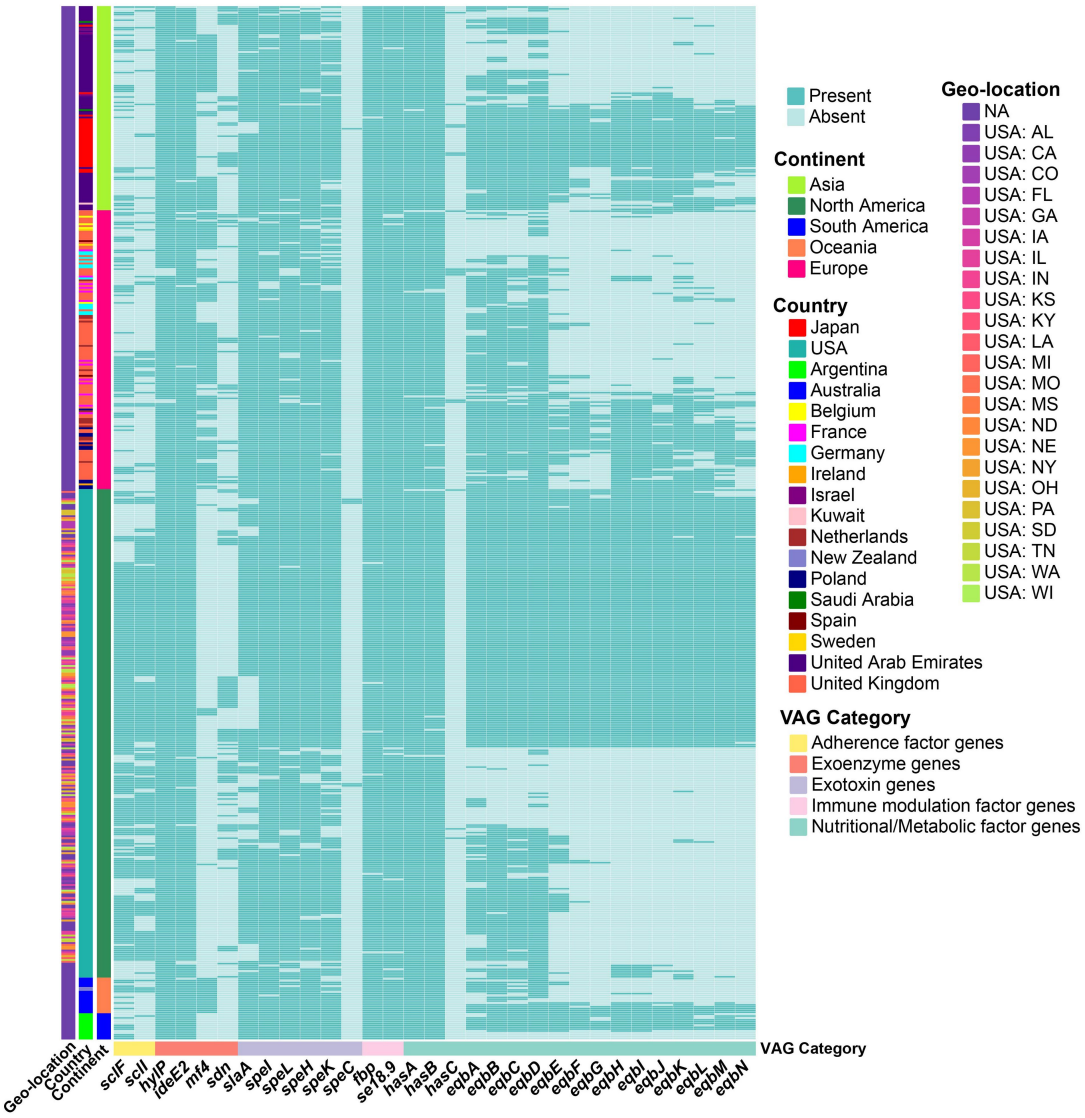
Heatmap showing the presence (green) or absence (light green) of accessory VAGs in *S*. *equi* strains from different continents. Each row represents a strain, grouped by continent (left annotation), country, and geo-location (additional left annotations). Each column represents a VAG, annotated by functional category. Strains are clustered based on gene presence/absence profiles. Country and geo-location annotations indicate the specific country and, for USA strains, the state of origin.

Some accessory VAGs exhibited distinct geographic distribution patterns (Figure 3). The adherence factor genes *sclF* and *sclI* were found to be more present in strains from North America, detected in 76.2% (199/261) and 74.7% (195/261) of the strains, respectively. The exoenzyme gene *mf*4 was more present in Asia, Europe, and in all strains from Oceania, but less common in North America (2.7%, 7/261) and absent in South America (0%, 0/14). Meanwhile, the Phospholipase A_2_ toxin (PLA_2_) SlaA gene *slaA* was less prevalent in North American strains, 71.3% (186/261) strains, compared to over 90% presence in strains from the other four continents (Figure 2C). The pyrogenic mitogen SpeC encoding gene *speC*, which was found to be carried by prophage SF370.1 from *S*. *pyogenes* SF370 (31), was detected in five North American strains and one Asia strain. Among the strains analyzed, the *eqb* gene locus was present in a majority of North and South American strains (more than 50%), while its presence was lower in strains from other continents (below 40%). Conversely, 54 strains completely lacked the equibactin siderophore system (*eqbA–N*; Figures 2B, 3). Within the *eqb* gene locus, the *eqbA*-*D* genes exhibited higher conservation compared to *eqbE*-*N* (Figure 3).

### 3.4 ARGs in *S. equi*

No penicillin-binding protein (PBP) mutations or other *β*-lactam resistance determinants were detected, consistent with our finding of an overall lack of acquired ARGs in the analyzed strains. In one isolate from South America, resistance was fond against quaternary ammonium disinfectants, through the *qacG* gene.

### 3.5 Identification of the MGEs in *S. equi*

**Figure 4:**
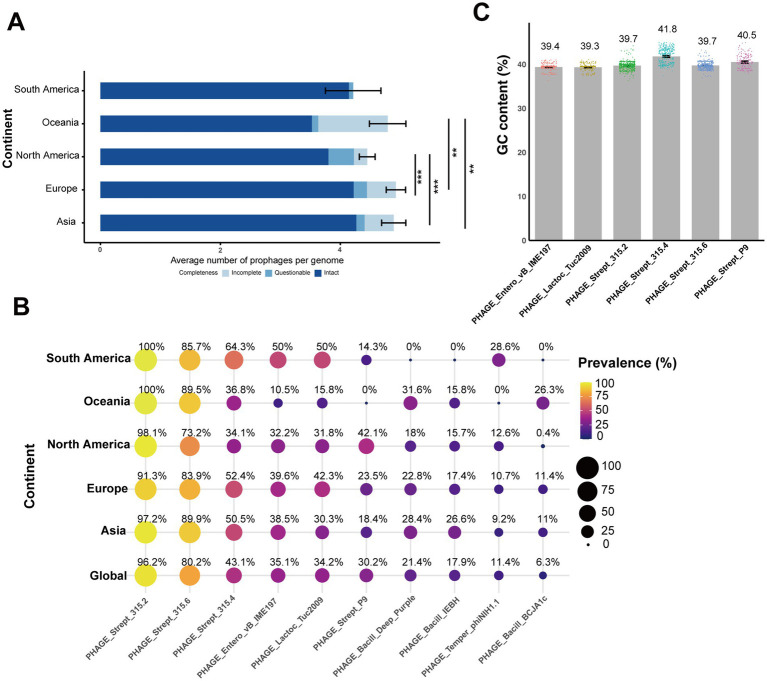
Diversity and distribution of prophages in *S*. *equi* across continents. **(A)** The integrity of prophages in *S*. *equi* from different continents. Stacked bar plots show the average number of intact, questionable, and incomplete prophages per genome for each continent. The average values are displayed as colored segments in each bar (blue: intact, light blue: questionable, gray blue: incomplete). **(B)** Bubble plot showing the prevalence of the top 10 prophages in *S*. *equi* strains across different continents and globally. Bubble size and color indicate the percentage of isolates carrying that prophage. Prevalence values are displayed inside each bubble. Prophages are ordered by their global prevalence. **(C)** Bar plot showing the average GC content (%) of the six most prevalent prophages in *S*. *equi* strains. Individual data points are shown as colored dots for each prophage. Bar charts display means with error bars representing 95% confidence intervals. Adjusted *p*-values were used to determine statistical significance, with thresholds defined as follows: *p <* 0.01 (**) and *p <* 0.001 (***).

**Figure 5:**
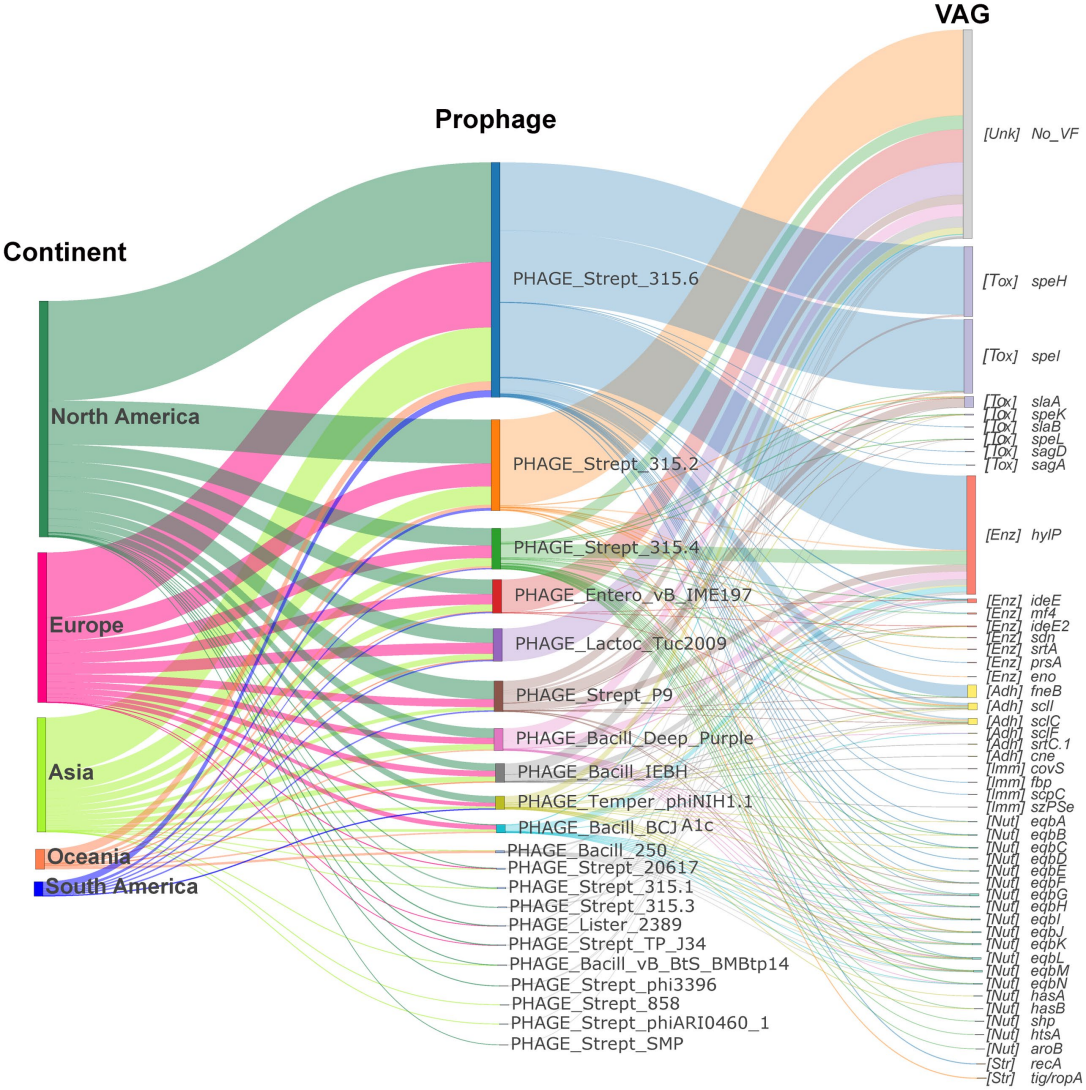
Contribution of the prophages to *S*. *equi* VAG distribution. Sankey diagram illustrating the prophage-encoding VAGs flow from continent to prophage to VAG. The left nodes represent continents, the middle nodes represent prophages, and the right nodes represent VAGs. Node and link colors indicate continent, prophage, and VAG category, respectively. VAG nodes are labeled with functional category abbreviations: Adh (Adherence factor gene), Enz (Exoenzyme gene), Tox (Exotoxin gene), Imm (Immune modulation gene), Inv (Invasion gene), Nut (Nutritional/Metabolic factor gene), Str (Stress survival gene).

Each of the 552 genomes contained 3 to 8 prophages, most of which were intact (Figure 4A). Pairwise comparisons showed that both North America and Oceania had significantly lower intact prophage counts compared to Asia (North America: *p* = 3.49e-07; Oceania: *p* = 0.0008) and Europe (North America: *p* = 2.58e-07; Oceania: *p* = 0.001; Figure 4A). In total, 21 different prophages were identified among the *S*. *equi* strains in our study (Figure 5). One PHAGE_Strept_315.2 was found in nearly all strains (531/552) across all continents (Asia: 97.2%, South America: 14/14, Oceania: 19/19, Europe: 91.3%, North America: 98.1%; Figure 4B). PHAGE_Strept_315.6 was the second most prevalent and present in 80.2% (443/552) of the strains. Other prophages were present in about 40% or lower of the strains (Figure 4B). South American strains exhibited the lowest prophage diversity (only seven high-frequency prophages) with PHAGE_Strept_315.2 and PHAGE_Strept_315.6 dominating (>85%, 14/14 and 12/14, respectively). Notably, the prophage PHAGE_Strept_P9 was detected at a higher frequency in North American strains (42.1%, 110/261) compared to the global average frequency (30.2%, 167/552; Figure 4B).

The mean GC content of these prophages (Figure 4C) ranged from 39.3 to 41.8%, with PHAGE_Strept_315.4 exhibiting the highest average GC content (41.8% ± 1.72), slightly exceeding the genomic background (41.3%). Other common prophages, including PHAGE_Strept_315.2, PHAGE_Strept_315.6, and PHAGE_Strept_P9, showed mean GC contents of approximately 39.7, 39.7, and 40.5%, respectively (Figure 4C), indicating consistent nucleotide composition within prophage populations in *S*. *equi*.

Global pairwise sequence comparisons showed that PHAGE_Strept_315.6, PHAGE_Strept_P9, and PHAGE_Strept_315.2 exhibited nucleotide identities greater than 70% with the well-characterized ΦSeq prophages ΦSeq4, ΦSeq3, and ΦSeq2, respectively, suggesting these three prophages potentially correspond to ΦSeq4, ΦSeq3, and ΦSeq2 previously described in *S*. *equi* (5).

**Figure 6:**
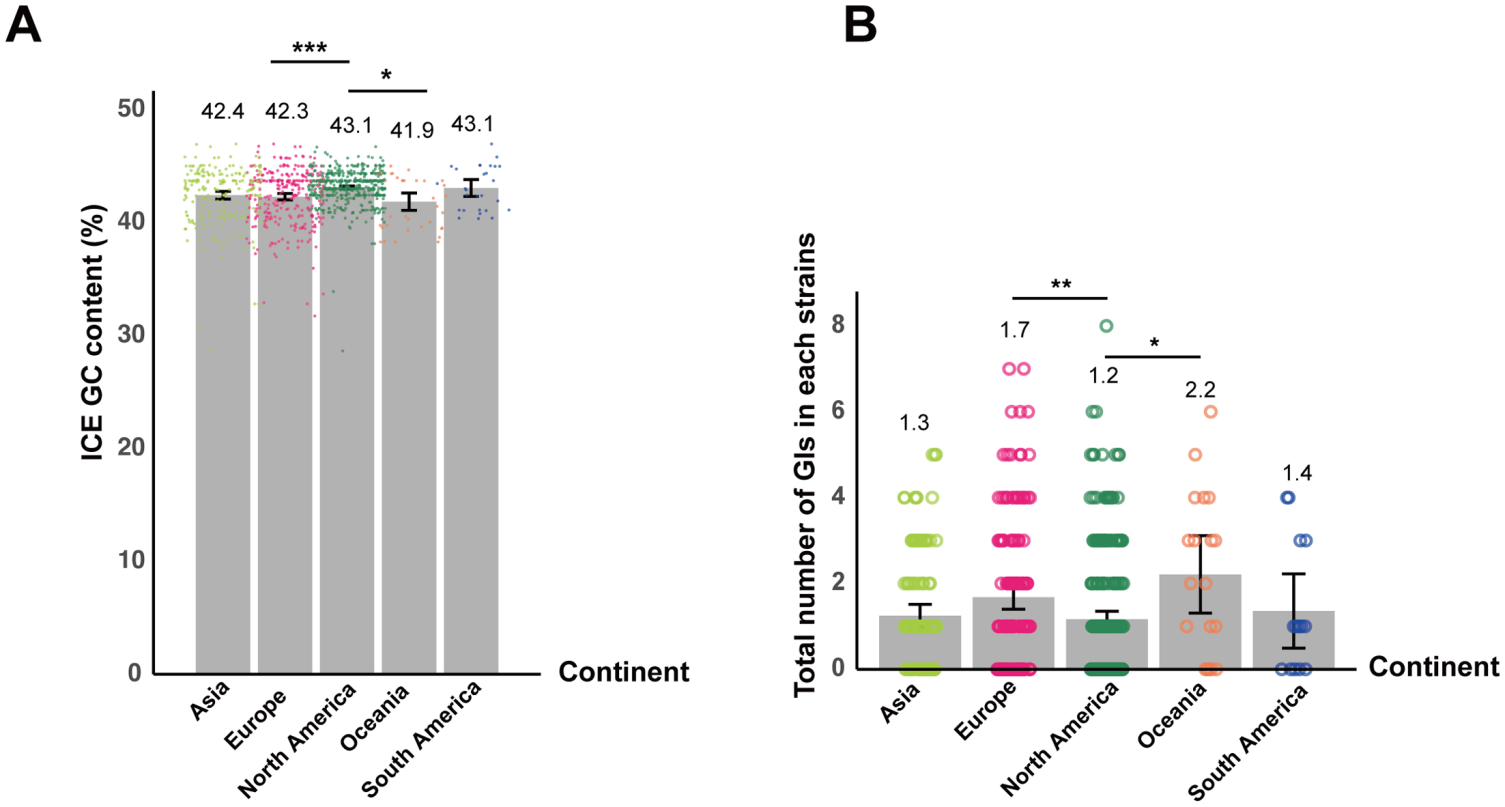
Geographic variation in ICE GC content and GI abundance among *S*. *equi* strains. **(A)** Bar plot showing the mean GC content of ICEs in *S*. *equi* strains from different continents. Individual data points are shown as colored dots. **(B)** Bar plot showing the average number of GIs per *S*. *equi* strain across different continents. Individual data points are shown as colored dots. Bar charts display means with error bars representing 95% confidence intervals. Adjusted *p*-values were used to determine statistical significance, with thresholds defined as follows: *p* < 0.05 (*), *p <* 0.01 (**), and *p <* 0.001 (***).

No intact or partial IMEs were detected across the entire dataset. However, the results of ICEscreen (v1.3.2) showed that all strains harbored two partial ICEs classified as type IV secretion system-type (T4SS-type) based on the conserved presence of VirB4, an ATPase essential for T4SS assembly and function. BLAST comparisons against all known ICE sequences from the ICEberg database (v2.0) (26) revealed that only four strains AQSNo.6 (Asia), AQSNo.12 (Asia), PA17110CY230005 (North America), and Pl0969 (Europe) exhibited ICE sequences with homology (>70% identity) to ICE*se*2, which encodes for a non-ribosomal peptide synthetase system in *S*. *equi* (32). In contrast, the remaining strains did not contain any homologous ICEs that were in the ICEberg database. To further validate the presence of ICE*Se*2, an additional screening was performed using ICEfinder2 (v2.0) directly. However, consistent with the initial analyses, ICEfinder2 did not identify any ICE*Se*2 elements among the strains (data not shown). The GC content of ICE-associated sequences was also examined. The average GC content of ICE sequences varied slightly among the geographic regions, ranging from 41.9% in Oceania to 43.1% in North and South America (Figure 6A). Notably, these ICE GC contents are slightly higher than the average GC content of the 552 *S*. *equi* genomes (41.3%). Pairwise comparisons showed that ICEs in strains from North America exhibited significantly higher GC content compared to those from both Europe (*p* = 0.0004) and Oceania (*p* = 0.04), indicating geographic variation in ICE nucleotide composition (Figure 6A).

GIs were detected in 319 of the 552 *S*. *equi* strains, with each draft genome harboring 0–8 GIs (Figure 6B). The average number of GIs per genome varied among continents. Strains from North America exhibited significantly lower mean GI count than Oceania (*p* = 0.04) and Europe (*p* = 0.003, respectively; Figure 6B).

### 3.6 Contribution of the MGEs to *S. equi* VAG distributions

To understand the mechanisms underlying the observed VAG distribution, the contribution of the MGEs to *S*. *equi* VAG distribution was investigated. It revealed that prophages, ICEs and GIs serve as primary carriers of VAGs in *S*. *equi*.

Although PHAGE_Strept_315.2 was the most common prophage, it did not always carry the highest number of VAGs, whereas PHAGE_Strept_315.6, PHAGE_Strept_315.4, and PHAGE_Strept_P9 were the dominant VAG carriers despite their lower prevalence (Figure 4B; Supplementary Figure S1). As shown in Figure 5, prophage-encoding VAGs predominantly comprised exotoxin genes, exoenzyme genes and adhesion genes. The hyaluronidase gene *hylP* was carried by various prophages, predominantly PHAGE_Strept_315.6, PHAGE_Strept_315.4, PHAGE_Strept_P9, PHAGE_Bacill_Deep_Purple, PHAGE_Bacill_IEBH and PHAGE_Bacill_BCJA1c (Figure 5). PHAGE_Strept_315.6 primarily carried the superantigen genes *speI*/*speH* and *hylP* (Supplementary Figure S1; Figure 5) while PHAGE_Strept_P9 carried the exotoxin gene *slaA* (Supplementary Figure S1).

**Figure 7:**
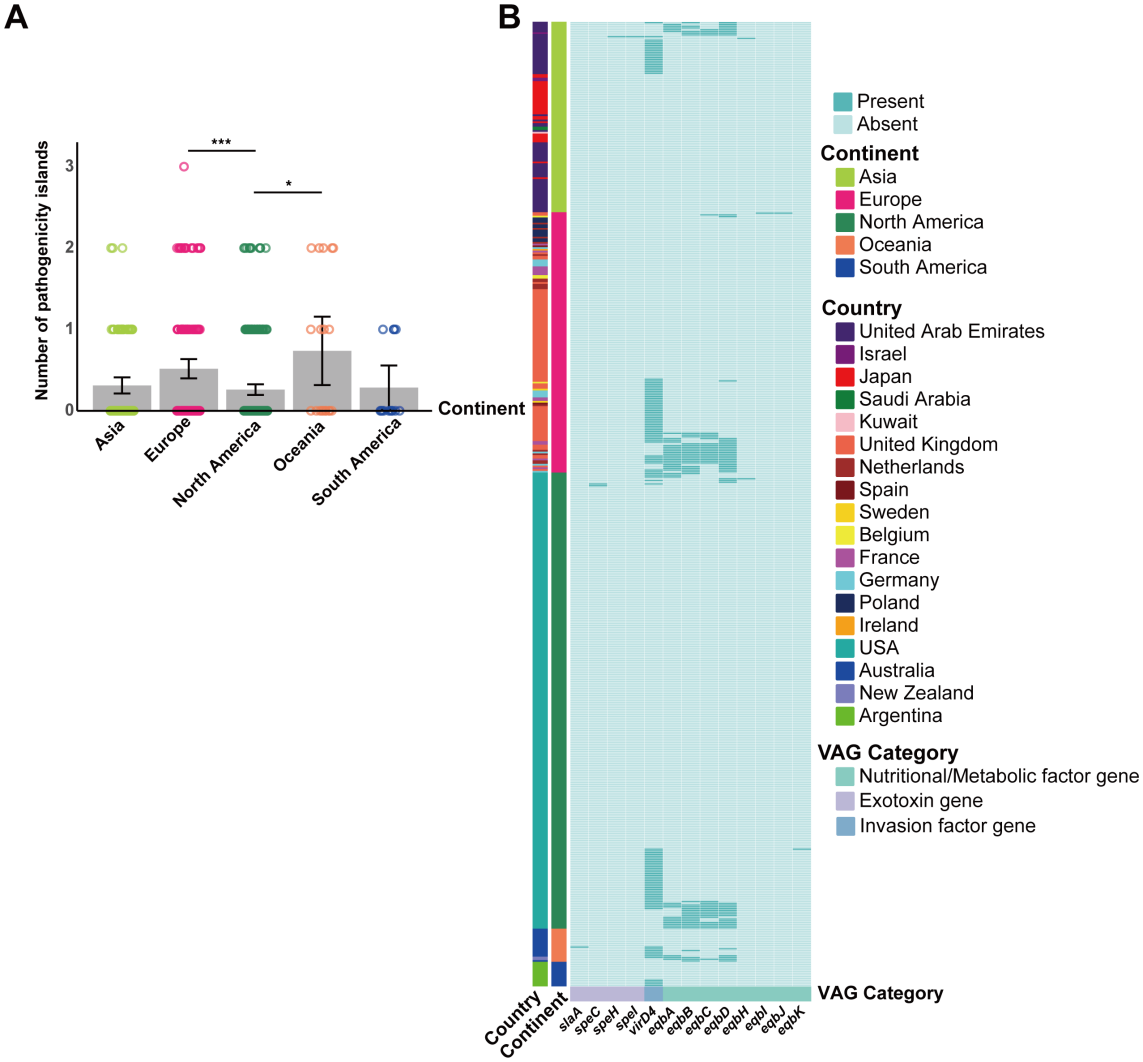
Contribution of GIs to *S*. *equi* VAG distribution. **(A)** Bar plot showing the average number of pathogenicity islands per *S*. *equi* strain across different continents. Individual data points are shown as colored dots. **(B)** Heatmap showing the presence (green) or absence (light green) of VAGs encoded by GIs in *S*. *equi* strains from different continents. Each row represents a strain, grouped by continent (left annotation). Each column represents a VAG, annotated by functional category. Strains are clustered based on gene presence/absence profiles.

Significant differences were observed in the number of pathogenicity islands between continents (*p* = 0.0024), with North America having significantly fewer pathogenicity islands compared to Europe and Oceania (adjusted *p* = 0.0006 and 0.0266, respectively; Figure 7). The main VAGs encoded by pathogenicity islands were *eqbA-D* and *virD4* (Figure 7).

**Figure 8:**
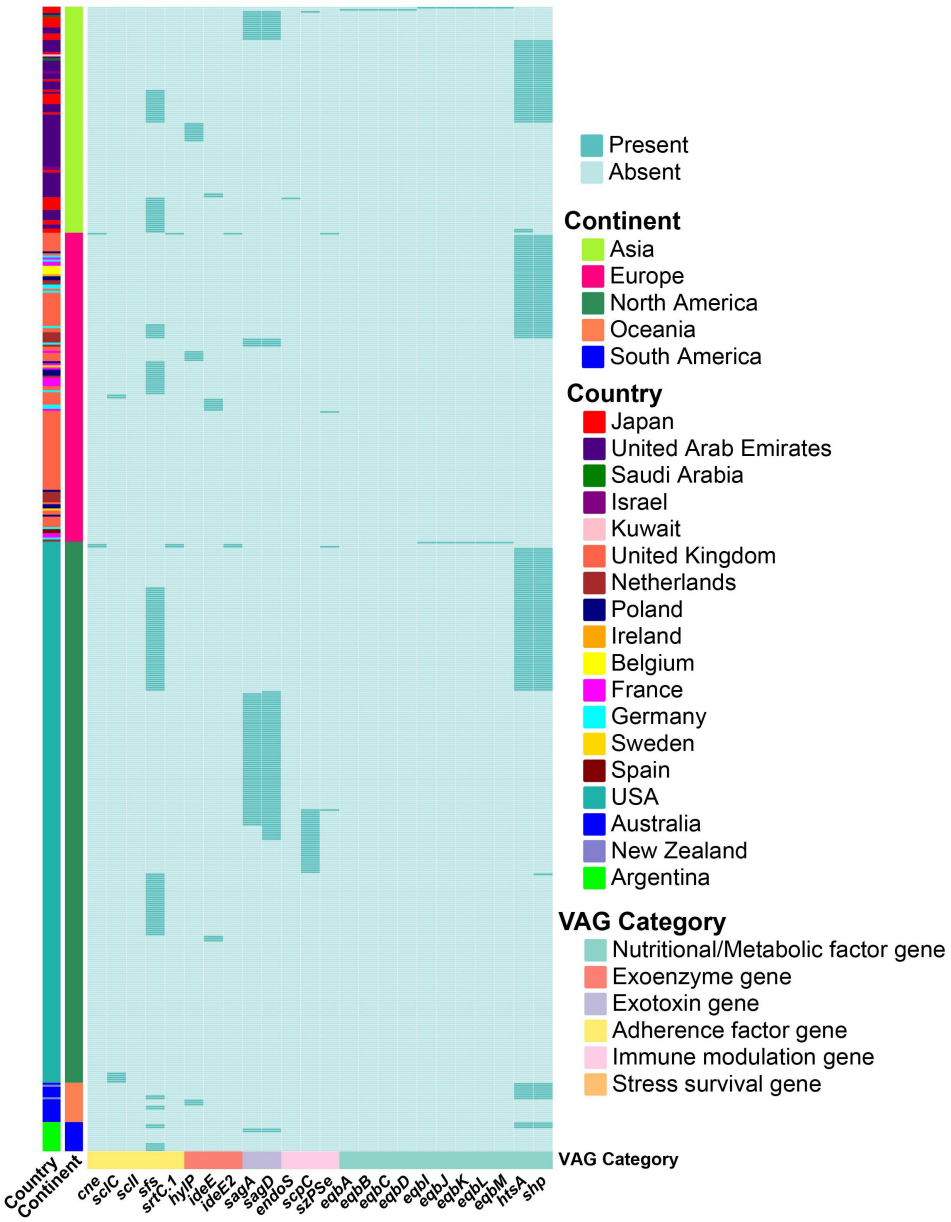
Contribution of the ICEs to *S*. *equi* VAG distribution. Heatmap showing the presence (green) or absence (light green) of VAGs encoded by ICEs in *S*. *equi* strains from different continents. Each row represents a strain, grouped by continent (left annotation). Each column represents a VAG, annotated by functional category. Strains are clustered based on gene presence/absence profiles.

The partial ICEs carried the *sfs* gene, which encodes a fibronectin-binding protein (Fn-binding protein). ICEs also carried streptolysin S-associated genes, *sagA* and *sagD*, mainly in strains from North America. Nutritional and metabolic factor genes *htsA* and *shp*, responsible for heme acquisition and iron uptake, were core genes in *S*. *equi* (Figure 2C), though some were also carried by ICEs (Figure 8).

## 4 Discussion

Our study indicates that *S*. *equi* exhibits a closed pangenome structure, typical for host-adapted bacteria like *Bordetella parapertussis* (*B*. *parapertussis*), *Bordetella pertussis* (*B*. *pertussis*), as well as all mycoplasma species as the extreme example of genome reductive evolution, which purge non-essential MGEs while retaining niche-critical genes (33, 34). Our findings provide evidence that host-adapted bacteria undergo genome streamlining, resulting in smaller and more clonal genomes (6, 35, 36).

Some accessory VAGs, which are also present in the Strangvac vaccine, exhibit significant geographic variation. This regional divergence is critical because vaccines impose selective pressure on bacteria, particularly in host-restricted species (37). Such selective pressure can drive the emergence of vaccine escape variants, as observed with serotype replacement and capsule switching of vaccine serotypes to nonvaccine types (NVTs) in *S*. *pneumoniae* following vaccination (38–40). These observations highlight a potential blind spot in current *S*. *equi* surveillance strategies, which often overlook the dynamic accessory genome (6, 41). Therefore, integrating the accessory genome analysis is necessary for proper surveillance of pathogens. It will enable the assessment of the efficacy of the antigens in vaccines and the early detection of potential vaccine escape variants.

Experimentally validated virulence factors of *S*. *equi* critical for pathogenicity include immune evasion (SeM, Se18.9, SzPse, slaA, superantigens speL and speK, EAG) (6, 9, 42–47), antibody disruption (ideE and ideE2) (48, 49), adhesion (CNE, FNEB, and SclC) (7, 10, 50, 51), capsule formation (hasA, hasB, and hasC) (52–54) and iron acquisition (eqb cluster) (32). Genes encoding CNE, SzPse, FNEB, SclC, EAG, ideE were identified as core VAGs that are universally conserved across the 552 strains. Although genes encoding SeM, Se18.9, superantigens SpeL, SpeK, SpeH and SpeI, as well as capsule biosynthesis genes *hasA* and *hasB* were classified as accessory VAGs in the present study, they showed broad conservation across all continents. Only genes encoding SlaA and the iron acquisition cluster *eqb* displayed notable geographical differences. This finding supports the genome streamlining hypothesis that host-adapted bacteria lose unnecessary genes over time, resulting in smaller, more clonal genomes that retain only essential virulence and fitness genes for specialized niches (35).

*speL*, *speK*, *speH* and *speI* encode superantigens that cause nonspecific T cell activation and release of proinflammatory cytokines, playing essential roles in severe streptococcal infections (55). Such immune overreaction is linked to streptococcal toxic shock syndrome (STSS) caused by *S*. *pyogenes* in humans (56). These exotoxins also contribute to the pathogenicity in *S*. *equi* and *S*. *zooepidemicus* (57, 58). Our study revealed that *speL*, *speK*, *speH*, and *speI* were widely distributed across *S*. *equi* strains, indicating that they are critical for the immune evasion strategies employed during *S*. *equi* infections. Furthermore, T cell receptor-binding deficient superantigens have shown promise as vaccine adjuvants by enhancing antigen presentation (59). These findings suggest that the conserved superantigen-encoding genes may serve as potential antigenic targets for the development of a strangles vaccine. However, the role of superantigens in the pathogenesis of *S*. *equi* remains largely unknown, and experimental validation is required to assess their immunogenicity and protective efficacy.

*slaA* and *slaB* are two homologous phospholipase A2 (PLA2) toxins encoding genes, found in both *S*. *equi* and *S*. *zooepidemicus*. *slaB* has been shown to be present in all strains of both species, while *slaA* is only in 31% of *S*. *zooepidemicus* strains and in all *S*. *equi* strains (26/26) (5). However, our results showed *slaA* was less prevalent in North American strains and more conserved in strains from other continents. PLA2 toxins were shown to be non-essential in the pathogenesis of strangles in a susceptible natural host (46). However, they can disrupt host cell membranes (Type II exotoxins) and their downregulation could mitigate excessive host tissue damage, helping *S*. *equi* evade immune clearance and promote long-term colonization (60). Persistent *S*. *equi* strains often lose some virulence genes, which may be part of an adaptive genomic streamlining process during chronic infection (6). Consistent with this, the lower conservation of the *slaA* gene in North American strains in our study suggests such an adaptation may occur in this region.

*Streptococcus* *equi* are almost always encapsulated. Non-encapsulated mutants have been shown to be less virulent (52, 53). Capsule synthesis is controlled by *hasA* (hyaluronate synthase), *hasB* (UDP-glucose dehydrogenase) and *hasC* (UDP-glucose pyrophosphorylase). While deletions in either *hasA* or *hasB* result in loss of capsule synthesis and virulence, deletions in *has*C do not lead to capsule loss (54, 61). Our findings showed that *hasA* and *hasB* were present in nearly all strains, whereas *hasC* was rarely present, providing further support for genome reduction in host-restricted pathogens.

The iron uptake systems of *S*. *equi* facilitate the development of lymph node abscesses (6, 32). Among these systems, equibactin (EqbA-N), a siderophore produced via the Nonribosomal peptide synthetases (NRPS), plays a major role (32, 62). However, our results showed that this gene cluster was absent in a multitude of strains. This finding aligned with previous reports of microevolution in persistently infected guttural pouches, characterized by deletions or amplifications, including the loss of the entire equibactin locus in some *S*. *equi* subpopulations (6). Large genomic deletions are hallmark indicators of genome reduction as bacteria adapt to more restricted niches (63–65). This reductive evolution involves shedding non-essential genes, which may include those encoding products analogous to host molecules that bacteria can exploit (66). Our results revealed that although the equibactin locus was absent in many strains of *S*. *equi*, the heme acquisition genes (*shp*, *shr*, *htsA*) were core genes. The results suggested that in strains lacking the equibactin locus, the core heme system may compensate for iron uptake, reflecting adaptation to fluctuating iron availability during infection likely due to changing niches in the respiratory tract. Similar findings of siderophore production dynamics have been observed during *B*. *pertussis* infection in mice (67). The coexistence of multiple iron acquisition systems not only compensates for variable iron availability but also enhances pathogenic fitness, as demonstrated in *Klebsiella pneumoniae* (*K*. *pneumoniae*), where the classical *K*. *pneumoniae* (cKp) strains typically encode genes for 1–2 siderophores, while hypervirulent *K*. *pneumoniae* (hvKp) strains encode genes for four siderophores (enterobactin, yersiniabactin, salmochelin, aerobactin), enhancing their superior tissue dissemination and hypervirulence (68). Whether this also applies to *S*. *equi* remains to be determined.

No acquired ARGs were detected, except for a resistance gene *qacG* against the quaternary ammonium compounds in a single South American strain (Arg0107). This absence of ARGs is unexpected, given the widespread resistance reported in other equine bacteria (69). However, a low prevalence of acquired ARGs is also observed in other highly specialized veterinary pathogens, such as *Mycoplasmata* spp. and *Actinobacillus pleuropneumoniae* (70, 71), suggesting that the specific lifestyle of pathogen may limit its capacity for horizontal gene transfer. As an obligate equine pathogen, *S*. *equi* has a reduced number of pili and other sortase-processed proteins on its surface compared to opportunistic pathogens like *S*. *zooepidemicus*, which may restrict the number of niches it can occupy (5, 72). This niche restriction, combined with its colonization pattern that avoids the nasopharynx where microbial exchange is common, further isolates *S*. *equi* from the broader bacterial gene pool, thus restricting its interaction with other bacteria and the subsequent acquisition of ARGs (73). Further microbiome studies are necessary to validate this hypothesis. Further microbiome studies are warranted to validate this hypothesis. It should also be considered that alternative mechanisms may contribute to the observed phenomena. Given the seemingly genome reduction, a decreased capacity for acquiring foreign DNA might also play a role. These possibilities require further investigation.

MGEs play a significant role in horizontal gene transfer (HGT) that leads to the dissemination of ARGs and VAGs and shapes the genome, often conferring selective advantages (74–78). Our study revealed that prophages, GIs, and ICEs play only a role in VAG distribution but not in ARG dissemination in *S*. *equi*, indicating that AMR does not pose a selective advantage for this bacterium. This finding may be explained by the unique lifestyle of the *S*. *equi*, whereby it hides in abscesses as well as in the guttural pouches where the diffusion of antibiotics is minimal.

A widespread prevalence of intact prophages was observed across all strains. They frequently carry superantigen genes (*speH*, *speI*) and the hyaluronate lyase encoding gene *hylP*. Specifically, prophages PHAGE_Strept_315.6, PHAGE_Strept_315.4 and PHAGE_Strept_P9 served as primary reservoirs that share a close relationship with phages of *S*. *pyogenes* (79, 80). Our analysis revealed that the GC content of common prophages closely matches the overall GC content of *S*. *equi* genomes, indicating that these prophages are well-adapted to *S*. *equi* and have been stable components of the bacterial genome. This finding is consistent with the role of bacteriophages as major elements in shaping *S*. *equi* evolution away from its ancestor, *S*. *zooepidemicus* (79).

While previous studies have confirmed the conserved insertion sites of ΦSeq1-4 and ICE*Se*2 (5, 32, 81, 82), no evidence of these elements were detected in the present study. Although prophages corresponding to ΦSeq2, ΦSeq3, and ΦSeq4 were identified, their carriage of key VAGs like *slaA*, *speK*, and *speL* showed a mosaic pattern inconsistent with previous reports. For example, the *slaA* gene was predominantly found on PHAGE_Strept_P9 (ΦSeq3-like prophage), contrary to its known association with ΦSeq2 (73). However, these discrepancies may be caused by the problematic assemblies of short-read sequencing of regions with lots of repeats, which are typical around prophages and ICEs. These elements were potentially distributed across multiple contigs. It may also cause failures to identify MGEs if hallmark genes used for their detection are deleted, rearranged, or mutated (83). Therefore, whole-genome sequencing approaches incorporating long-read data, closed genomes, or targeted PCR validation are needed to reconcile these observations conclusively.

In conclusion, our analysis of 552 global *S*. *equi* genomes provides a comprehensive framework for understanding its accessory genome and genome adaptation. While acquired antibiotic resistance remains absent, geographic variations in MGEs and VAGs were observed. Our findings support the concept of “genome streamlining,” where this host-adapted pathogen sheds non-essential genes while retaining specialized genes. The geographic patterns likely arise from complex interactions involving host adaptation, and the ecological factors underlying these patterns remain to be determined. Future studies employing functional genomics, and long-read sequencing data will be necessary to clarify these dynamics. Our work provides a high-resolution map of *S*. *equi* VAGs, which is crucial for future vaccine strategies. While core antigens remain primary targets, the observed regional variations in key VAGs highlight the importance of ongoing genomic surveillance to monitor antigenic drift and inform the timely update of vaccine formulations (Table 1), thereby maintaining efficacy against evolving local pathogen populations.

**Table 1:** Potential vaccine antigens to be included in a regional vaccine.

Genes (Antigens)	Brief function	Regions
*fbp* (SeM)	M-like protein, adhesion (42, 43)	Globally conserved
*ideE* (IdeE), *ideE2* (IdeE2)	IgG-degrading enzymes (48, 49)	
*cne* (CNE), *sclC* (SclC)	Adhesion proteins (8, 10)	
*eag* (EAG)	Immunoglobulin G binding protein (9)	
*hasA* (hasA), *hasB* (hasB)	Hyaluronic acid capsule synthesis (54)	
*speH* (SpeH), *speI* (SpeI), *speK* (SpeK), *speL* (SpeL)	Superantigens (55)	
*sclF* (SclF), *sclI* (SclI)	Adhesion (collagen-like proteins) (10)	More prevalent in North America
*mf4* (Mitogenic factor4)	A mitogenic endonuclease and novel streptococcal superantigen (84)	Absent in North and South America
*slaA* (SlaA)	Phospholipase A2 toxin (5)	Less prevalent in North America
*eqb* cluster (EqbA-N)	Iron acquisition (siderophore) (32)	More prevalent in North America

*Listed antigens are based on genomic data in the present study, which includes both well-conserved proteins already used in current strangles vaccines and newly proposed candidates.

## Data Availability

The datasets presented in this study can be found in online repositories. The names of the repository/repositories and accession number(s) can be found in the article/Supplementary material.

## Footnotes

1
http://www.mgc.ac.cn/VFs/

2
https://github.com/tseemann/abricate

3 https://www.chiplot.online/
